# Improved Flux Performance in Brackish Water Reverse Osmosis Membranes by Modification with ZnO Nanoparticles and Interphase Polymerization

**DOI:** 10.3390/membranes14100207

**Published:** 2024-09-27

**Authors:** Jesús Álvarez-Sánchez, Germán Eduardo Dévora-Isiordia, Claudia Muro, Yedidia Villegas-Peralta, Reyna Guadalupe Sánchez-Duarte, Patricia Guadalupe Torres-Valenzuela, Sergio Pérez-Sicairos

**Affiliations:** 1 Departamento de Ciencias del Agua y Medio Ambiente, Instituto Tecnológico de Sonora, 5 de Febrero 818 Sur, Ciudad Obregón 85000, Mexico; yedidia.villegas@itson.edu.mx (Y.V.-P.); reyna.sanchez@itson.edu.mx (R.G.S.-D.); lupitatorreses@hotmail.com (P.G.T.-V.); 2 Tecnológico Nacional de México, Instituto Tecnológico de Toluca, Avenida Tecnológico S/N Col, Metepec C.P. 52140, Mexico; cmurou@toluca.tecnm.mx; 3 Center for Graduates and Research in Chemistry, National Technological Institute of Mexico Technological Institute of Tijuana, Tijuana 22000, Mexico; sergio.perez@tectijuana.edu.mx

**Keywords:** concentration polarization, desalination, interfacial polymerization, reverse osmosis, TFC membranes, ZnO nanoparticle

## Abstract

With each passing year, water scarcity in the world is increasing, drying up rivers, lakes, and dams. Reverse osmosis technology is a very viable alternative which helps to reduce water shortages. One of the challenges is to make the process more efficient, and this can be achieved by improving the capacity by adapting membranes with nanomaterials in order to increase the permeate flux without exceeding the limits established in the process. In this research, brackish water membranes (BW30) were modified with ZnO nanoparticles by interphase polymerization. The modified membranes and BW30 (unmodified) were characterized by FTIR, AFM, contact angle, and micrometer. The membranes were tested in a cross-flow apparatus using 9000 ppm brackish water, and their permeate flux, salt rejection, and concentration polarization were determined. The salt rejection for the 10 mg ZnO NP membrane was 97.13 and 97.77% at 20 and 30 Hz, respectively, sufficient to generate drinking water. It obtained the best permeate flux of 12.2% compared to the BW30 membrane with 122.63 L m^−2^ h^−1^ at 6.24 MPa and 30 Hz, under these conditions, and the concentration polarization increased.

## 1. Introduction

**Figure 1:**
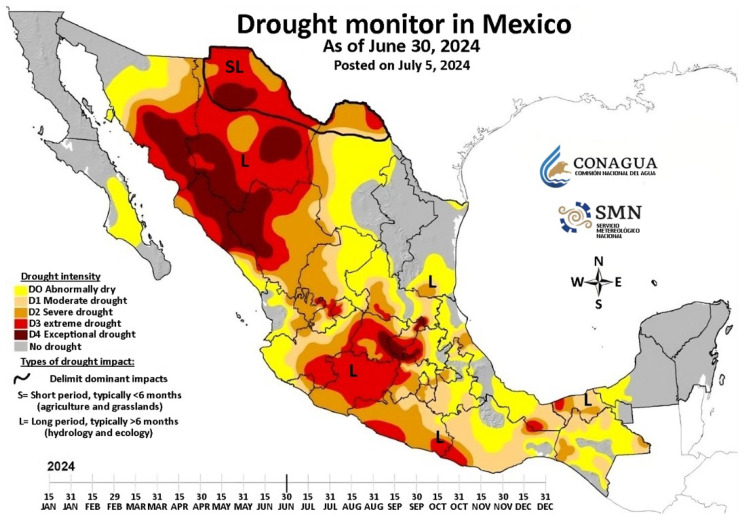
Mexico Drought Monitor by CONAGUA, 30 June 2024 [4].

Water is at the epicenter of sustainable development and is fundamental to socioeconomic development, energy, food production, ecosystems, and human survival. Water is also a crucial part of climate change adaptation and is a critical link between society and the environment [1,2]. Water-scarce countries must implement effective water resource management, based on potential solutions such as building water conservation facilities, virtual water trading, and improving water use efficiency and recycling [3]. By 2024, most of the Mexican territory suffered from drought (Figure 1), ranging from exceptional drought to moderate drought, and most of the states of Sonora, Chihuahua, Durango, and Sinaloa are affected by exceptional drought and extreme drought [4]. In the state of Sonora, the Alvaro Obregón dam has a water reserve of 10% for July 10 of the present year, and the dam is the main source of supply for the municipality of Cajeme and surrounding towns that supply local industries, ranchers, and farmers [5]. Therefore, it is important to seek solutions to mitigate, reduce, or be auxiliary sources during an extreme drought. Desalination is an alternative solution for water shortage problems [2], and the reverse osmosis process is a technology that implements semi-permeable membranes by applying a hydraulic pressure greater than the osmotic pressure on a solution of higher salt concentration to obtain a permeate flow of low salt concentration [6,7]. Pfeffer, Traube, and others studied osmotic phenomena with ceramic membranes in the 1850s. In 1931, the process was patented as a water desalination method and was given the name reverse osmosis [7]. Reid and Breton, in 1959, succeeded in synthesizing a cellulose membrane with a salt rejection of 98% but with a low permeate flux of 0.8 µL h^−1^ cm^−2^ atm^−1^ and a very high pressure of 6.5 MPa. Loeb-Sourirajan, in 1963, prepared an anisotropic cellulose membrane with a flux greater than 10 times that of Breton membranes and with very similar salt rejection. In 1985, Cadotte developed the interphase polymerization method to produce composite membranes [7], which consists of reacting bifunctional or trifunctional molecules to make a polycondensation reaction by functional groups such as -NH_2_, OC-Cl, -OH y -COOH, and the polymerization occurs at the interface of the organic solution and the aqueous solution, hence the name [8,9].

**Figure 2:**
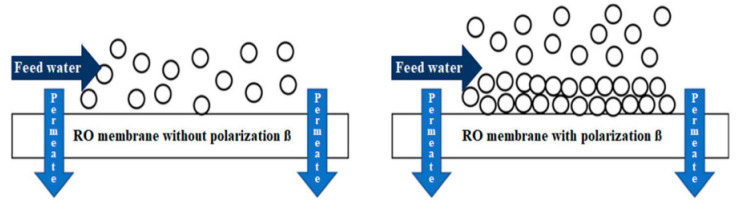
Concentration polarization in reverse osmosis membrane [10].

Composite membranes are affected by a transport phenomenon called concentration polarization (Figure 2), which implies that the feed concentration near the membrane increases [10] due to salt accumulation, causing a scaling that affects RO process performance, decreasing salt rejection, lowering permeate quality, and increasing process operating pressure [11,12]. Kucera (2015) considered that concentration polarization of 1.2 is the maximum allowable limit value for a membrane not to polarize so fast and not to have salt scaling [12]. When the suggested ranges of operating pressure and concentration polarization are not considered, problems have been reported in the industry ranging from partial or total clogging of the membranes resulting in water production process stoppages to accidents due to lack of knowledge of reverse osmosis process control.

The cost of desalinating seawater with reverse osmosis technology is 0.78 USD $/m^3^ [13]. If improvements are made in some part of the process, e.g., in the membranes by making them more efficient in generating higher permeate flux while maintaining permeate quality, the cost per m^3^ produced can be reduced [14]. Istirokhatun (2022) elaborated on Ag nanobars then integrated by interphase polymerization on the membrane surface; the nano bars served as sacrificial precursor materials to leave nano spaces and generate Ag nanoparticles. Upon testing the modified membranes, the water permeability was 10.4 L m^−2^ h^−1^ bar^−1^, more than twice that of the dense polyamide (PA) membrane, which was 4.5 L m^−2^ h^−1^ bar^−1^ [15]. Rebiee (2015) used different concentrations from 0.3 to 4% of ZnO nanoparticles to dope polyvinyl chloride ultrafiltration membranes; these membranes were prepared by the phase inversion method. ZnO NP caused an increase in the permeate flux of the membranes from 213 L m^−2^ h^−1^ for the unmodified PVC membrane to 402 L m^−2^ h^−1^ at 3% by weight for the ZnO/PVC membrane [16]. Ayyura et al. (2020) prepared membranes from polyvinylidene fluoride (PVDF) doped with ZnO nanoparticles through the phase inversion method, where they obtained a permeate flux of 170.73 L m^−2^ h^−1^ with a concentration of 0.2% ZnO nanoparticles [17]. Isawi, in 2016, firstly modified membranes using interphase polymerization with trimesoyl chloride (TMC) and m-phenylenediamine (MPD); secondly, free radical polymerization was used to graft poly (methacrylic acid) (polyMAA) onto polyamide (PA) membranes by means of the amide groups and carboxylic acids existing in the PA, in the same solution in which ZnO nanoparticles were added, and these reacted forming covalent bonds with the carboxylic acids of the polyMAA and also formed hydrogen bonds. The membranes were tested in the RO system with a solution of 2000 mg L^−1^ of NaCl and a pressure of 1.5 MPa. The membrane with the best performance with respect to flux had 0.1% ZnO nanoparticles; permeate flux was L m^−2^ h^−1^, and salt rejection was at 98% [18]. Therefore, the importance of using ZnO doping to improve the permeate flux of the reverse osmosis desalination process is highlighted.

Consequently, this research aims to modify thin-film composite membranes (TFC) to desalinate brackish water and increase the permeate flux through a polyamide layer and ZnO nanoparticles. In this research work, the BW30 (brackish water) membrane was modified by interphase polymerization by coating with a polyamide layer and doping with ZnO nanoparticles. The polyamide layer was made from TMC (1,3,5-Benzenetricarbonyl trichloride), and PP (piperazine). Three membranes were modified with ZnO nanoparticles. A membrane coated only with polyamide was also prepared as a control. BW30 control (polyamide layer only) membranes were characterized by AFM (atomic force microscopy), FTIR (infrared spectrophotometer), contact angle, and micrometer. The performance of the membranes was tested in the cross-flow equipment by determining permeate flux, salt rejection, and concentration polarization.

## 2. Materials and Methods

The chemicals used were as follows: ZnO nanoparticles with a diameter of less than 100 nm (St. Louis, MO, USA), piperazine (PIP with a purity of 99%, St. Louis, MO, USA); 1,3,5-Benzenetricarbonyl trichloride (TMC, 98%, St. Louis, MO, USA). All the reactives were purchased from Sigma-Aldrich. NaOH (97.0%; Jalmek, San Nicolás de los Garza, Nuevo León, México) and polivinyl alcohol (PVA, Selvol degree 205, 88% grade of hydrolysis, Pasadena, TX, USA) were also used for thin-film composite (TFC) membrane synthesis. Cyclohexane (99%; Fermont, Monterrey, Nuevo León, México) and deionized (DI) water were used for reverse osmosis membrane preparation. The commercial membrane BW30 (Dow Filmtec, Delfgauw, Netherlands) and the sea salt were supplied from Instant Ocean (St. Blacksburg, VA, USA).

### 2.1. Modification of BW30 Membranes with ZnO NP

**Figure 3:**
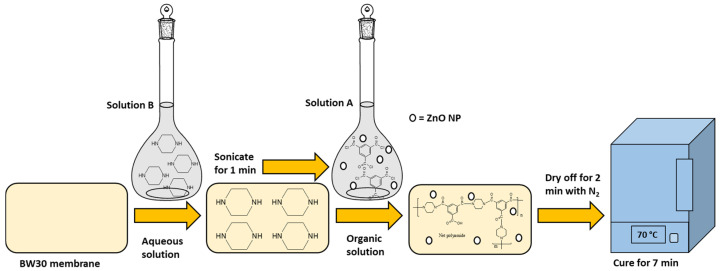
Procedure used to modify membranes with interfacial polymerization and ZnO NP [9].

An extra PA layer via interfacial polymerization was added to the RO membrane (BW30). The modifications were using 5 mg, 10 mg, and 15 mg per 100 mL DI water of ZnO NP (Table 1). 1 g of TMC was dissolved in 100 mL of cyclohexane (solution A) for the organic solution. For the aqueous solution, 0.25% *w*/*v* of PVA, 0.50% *w*/*v* of NaOH, and 0.25% *w*/*v* of PIP were dissolved in 100 mL of DI water (solution B) [8,9] (Figure 3). The control was the BW30 membrane modified with an extra PA layer without ZnO NP (Table 1).

The procedure used was the method previously reported by Torres-Valenzuela et al. (2022) [8] with some modifications, using ZnO nanoparticles and a BW30 membrane. Solution B was brushed on a 190 cm^2^ piece of BW30 membrane. Consequently, solution B was dispersed along the plane surface. Solution A was also applied and allowed to react for 60 s before being placed in an inert N_2_ atmosphere to finish the polycondensation reaction. The membrane was dried under 70 °C for 420 s and then washed to eliminate excess reagents or impurities.

### 2.2. Characterization of Modified Membranes

A contact angle analysis was conducted in Dataphysics equipment, model OCA 15EC, to determine the membranes’ hydrophilicity and hydrophobicity. One membrane piece of 3 cm × 3 cm was placed on the base of the equipment, and five drops of water were applied to the membrane at different sites on the surface with a distance of less than a centimeter between each measurement. The results were processed using SCA20 software version 2012 (Filderstadt, Baden-Württemberg, Germany).

The infrared spectroscopy Fourier transformed (FTIR) technique was carried out for the functional group identification. The equipment used was a Thermo Scientific, model Nicolet iS5, with an ATR accessory. One membrane piece of 1 cm × 1 cm was placed on the FTIR-ATR (Germanium crystal) with the PA face down. The data collected was processed using OMNIC software version 2012 (Waltham, MA, USA).

To know the roughness of the membrane, an atomic force microscope was used (AFM, model TT-AFM-WORKSHOP, Hilton Head Island, CA, USA). A piece of 1 cm × 1 cm membrane was glued to the equipment base, and the AFM 50 μm software version 2012 (Hilton Head Island, CA, USA) was started; the camera was focused, and a small tip (radios < 15 nm, material: single crystal silicon, length cantilever: 215–235 μm) was brought close to the sample, which carries out the analysis. For each sample of membrane, a triplicate test was performed using different scanning area sizes of 10 μm × 10 μm, 30 μm × 30 μm, and 50 μm × 50 μm. The size of the particles was measured using 2D images in the Gwyddion software version 2013 (Brno, Southern Moravia, Czech Republic).

Membrane thickness was measured using an analog micrometer (Mitutoyo, model 7300S, Kawasaki, Kanagawa, Japan). The average result was taken from seven measurements with an area of 5 cm × 12 cm. The analysis of SEM of the membranes was determined using the equipment expressed in the methodology of Torres-Valenzuela [8]. A VEGA3 Scanning Electron Microscope TESCAN (SEM, 25 kV, Brno, Southern Moravia, Czech Republic) was used, and before the SEM analysis, all samples were sputter-coated with gold (99.999%) at 16 mA for 4 min using a SPI-MODULE sputter coater (West Chester, PA, USA).

### 2.3. Membrane Performance Test

To evaluate the membranes’ performance, cross-flow equipment was used. The details of the systems were reported by Torres-Valenzuela et al. (2022) [8]. The main measure variables were permeate flux, permeate, rejection flow, salt rejection, and concentration of polarization. The membranes were hydrated for 24 h in deionized water before the different tests. One membrane per cell (3 in total) was placed in the cross-flow equipment, with the PA side facing the feed channel.

The equipment was filled with DI water, and the pump was turned on. The membranes were compacted for 1 h at 2.07 MPa. Then, the tank was fed with synthetic sea salt solution at 9000 ppm (Instant Ocean salt) in an operation range of 2.18 until 6.22 MPa. To quantify the performance of the membranes, two frequency ranges were used for the operation of the high-pressure pump, 20 Hz and 30 Hz. The cell had an active area of 42 cm^2^. The permeate flow was collected, and the rejection flow was recirculated to the feed tank. The permeate and rejection flow were measured regarding conductivity (YSI 30) and temperature. In all the treatments, the concentration of salt, permeate flow rate, volumetric permeate flux, rejected salt, and concentration polarization were calculated according to Equations (1)–(7) (Table 2), previously reported [12] in Dévora-Isiordia et al. (2023).

## 3. Results and Discussion

### 3.1. Characterization of BW30 Membrane, Control Membrane, and Membranes Modified with ZnO NP

**Figure 4:**
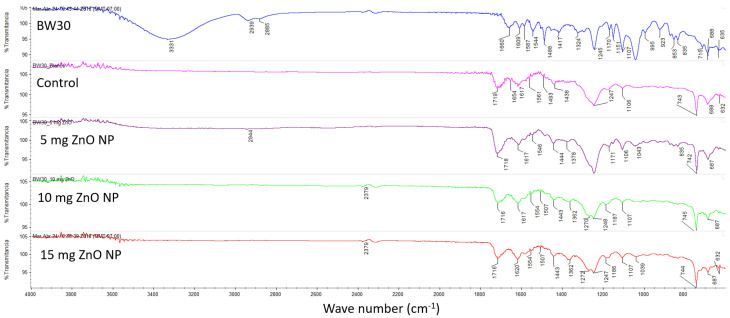
IR spectra of unmodified and modified membranes: BW30 (blue line), Control (pink line), 5 mg ZnO NP (purple line), 10 mg ZnO NP (green line), and 15 mg ZnO NP (red line).

Figure 4 shows the spectra obtained from the modified membranes by FTIR-ATR. In the BW30 membrane, a characteristic peak was found: 3331 cm^−1^ attributed to the N-H stretching vibration, 1660 cm^−1^ to the stretching of the -C=O group (strain, Amide II), 1587.08, 1543.64 and 1488. 27 cm^−1^ to the stretching vibration of the aromatic C-C ring, 1244.60 cm^−1^ to the asymmetric C-O-C stretching of the aryl ether group (Ar-O-Ar), and 1169.59 and 1151.22 cm^−1^ to the symmetric stretching of the sulfone functional group (Ar-SO_2_-Ar) [22,23,24,25].

**Figure 5:**
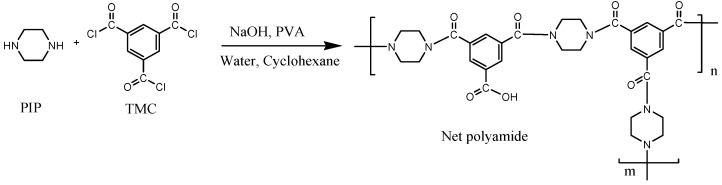
The reaction between piperazine and 1,3,5-Benzenetricarbonyl trichloride (TMC) to synthesize the net polyamide [8].

Table 3 summarizes the signals found in the membranes with their respective wave number. The interphase polymerization reaction between the acyl chloride groups and water produces the functional group -COOH [8,22,26], which was found in all the membranes with a peak near 1716 cm^−1^, with the exception of membrane BW30 because it was the membrane without modification (Figure 4). In all the spectra of the modified membranes, a peak appears around 1617 cm^−1^, attributed to the CO-NH bending vibration, which indicates that the characteristic bond of the polyamides was formed, based on the piperazine chemistry [24]. The structure of piperazine and the polymeric network of the polyamide can be seen in Figure 5.

**Figure 6:**
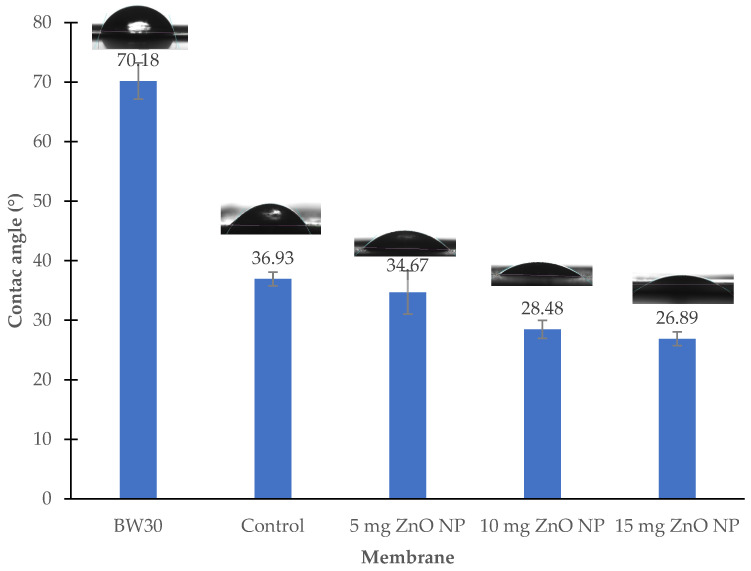
Membrane surface contact angle measurements.

Figure 6 shows the contact angle results of the unmodified BW30 membrane and the modified membranes. The unmodified BW30 membrane presented the highest contact angles with an average of 70.18 ± 3.05° but still within the range of having affinity to water, according to Isawi et al., 2016, and Perez-Sicairos et al., 2016 [9,18]. The standard deviation indicates that it is a reliable result, as it has a percentage of less than 10% with respect to the average; in fact, the standard deviation was less than 10% in all membranes, which indicates that they are reliable contact angle results (Figure 6). The membrane with 15 mg ZnO NP obtained the lowest contact angle at 26.89 ± 1.15°. The membrane with 15 ZnO NP was 61.7% more hydrophilic than the BW30 membrane, and the membrane with 15 mg ZnO NP was 27.2% more hydrophilic than the control membrane.

The addition of ZnO NP caused the membrane to acquire more hydrophilic characteristics than all other modified membranes due to the formation of intermolecular forces of hydrogen bridges between the hydrogen in water and the oxygen in zinc oxide [27]. The ratio of the amount of ZnO NP is inversely proportional to the contact angle, which is reflected in an increase in hydrophilicity [8,28]. Compared with what was reported by Perez-Sicairos et al. (2016) [9], these results presented the same behavior between contact angle and hydrophilicity.

Table 4 shows the roughness results at different scan areas (10, 30, and 50 µm), particle size, and thickness of the modified and unmodified membranes. The membranes become rougher simply by adding the new polyimide layer. It is also evident that adding ZnO NP makes the membranes even rougher. However, the membrane with 10 mg ZnO NP decreases the roughness with respect to the 10 and 50 µm scans, respectively. On the other hand, it can be observed that in the membrane with a concentration of 15 mg of ZnO NP, the roughness increases in the three scans [8]; this may indicate that the nanoparticles are agglomerating in such a way that the roughness increases due to the accumulation of particles.

**Figure 7:**
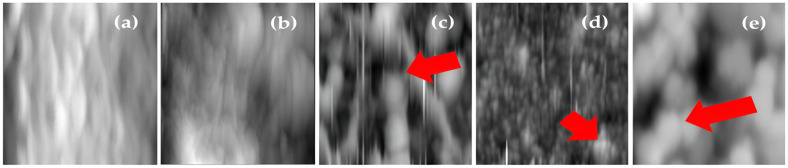
Analysis AFM 2D (10 × 10 µm): (**a**) BW30, (**b**) Control, (**c**) 5 mg ZnO NP, (**d**) 10 mg ZnO NP, and (**e**) 15 mg ZnO NP. The red arrows indicate an agglomerate of ZnO NP.

Figure 7 shows the 2D AFM analysis of the membranes with ZnO NP on their surface (Figure 7c–e), where it can be observed that the BW30 membrane and the control membrane show their surface as a polyamide network. The membranes with 5 mg of ZnO NP and 15 mg of ZnO NP present agglomerations of nanoparticles (See Figure 7d). The agglomeration of nanoparticles may be due to van der Waals forces of attraction, which causes their chemical reactivity to be reduced [29]. This is one of the main drawbacks of membrane modification, and a method is needed to make a uniform dispersion and distribution of nanoparticles within the polymer matrix [30].

**Figure 8:**
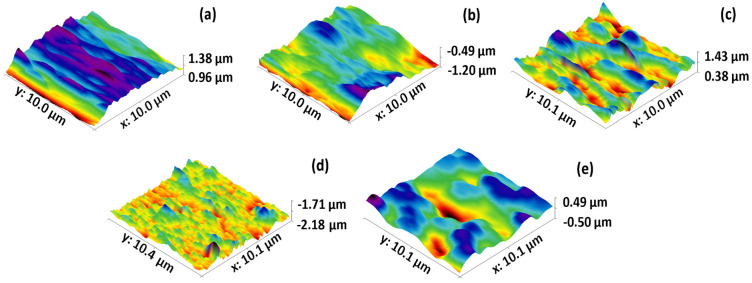
3D AFM results showing surface roughness for the tested membranes: (**a**) BW30, (**b**) Control, (**c**) 5 mg ZnO NP, (**d**) 10 mg ZnO NP, and (**e**) 15 mg ZnO NP. Scan area 10 × 10 µm.

Figure 8 shows the 3D results obtained from the ZnO NP modified membranes at a scanning area of 10 µm. In Figure 8c,e, corresponding to the 5 and 15 mg ZnO NP membranes, high points and purple colors can be observed up to very low points of reddish colors, in which the agglomerations of nanoparticles can be better appreciated. As for Figure 8d corresponding to the 10 mg ZnO NP membrane, it presents a more uniform roughness without agglomeration of nanoparticles. The variation of agglomerations on the surface of the membranes may be due to the variation of the given ultrasound time, just before emptying the solution with nanoparticles in the membrane preparation. Agglomeration and sedimentation of nanoparticles occur rapidly due to van der Waals forces. Thus, ultrasound is very effective in dispersing them to reduce the size of the agglomerates [31]. However, the sedimentation that occurred just after removing the ultrasound solution was very fast, so depending on the solution, the ultrasound times varied between membranes.

**Figure 9:**
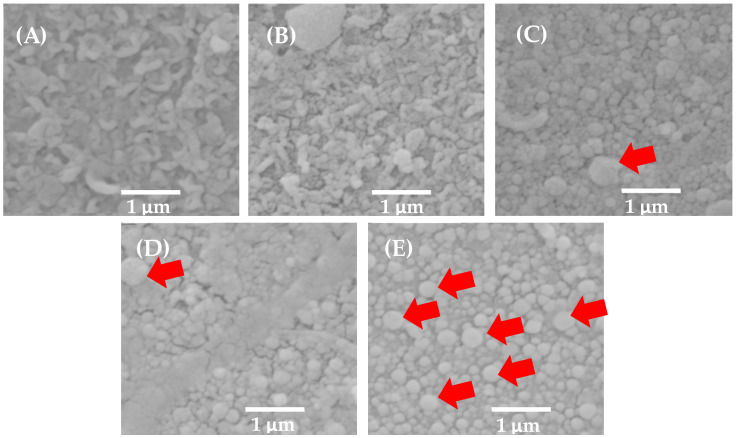
SEM images at 1 µm: (**A**) BW30 membrane, (**B**) control membrane, (**C**) 5 mg ZnO NP membrane, (**D**) 10 mg ZnO NP membrane and (**E**) 15 mg ZnO NP membrane. The red arrow indicates the agglomerates.

In Figure 9 SEM, micrographs indicate that in Figure 9C–E, there are nanoparticles on the surface of the modified membranes of spherical architecture. In the membrane with 10 mg ZnO NP in Figure 9D, the particle sizes are a little larger than 100 nm mostly, compared to the membrane with 5 mg ZnO NP. For the case of Figure 9E, we have particle sizes up to 375 nm (13 agglomerates) and a saturation of ZnO NP on the 15 mg ZnO NP membrane indicating agglomeration and aggregates of nanoparticles [18,32,33].

Commercial ZnO nanoparticles have an average particle diameter of less than 100 nm. In all micrographs, ZnO particles smaller than 100 nm are visible; in fact, they are the most abundant. In the 5 mg ZnO NP membrane, an agglomerate of approximately 500 nm and 5 agglomerates of approximately 350 nm are observed. This corroborates what was exposed in this research by the AFM analysis that also detected agglomerates of ZnO NP larger than 500 nm; both techniques complement each other. The challenge for new research is to make the nanoparticles distribute in their smallest particle size (without agglomerates) in order to obtain a better exploitation of their physical and chemical properties by using new membrane modification techniques and/or improving the existing ones.

### 3.2. Performance Test in Reverse Osmosis Membranes in an Equipment Cross Flow

The results obtained for pressure versus permeate flux of the reverse osmosis membranes at a concentration of 9000 ppm of synthetic sea salt at a pressure of 2.18 to 6.22 MPa are shown in Figure 9. The flux obtained at this concentration coincides with the results of the permeability and hydrophilicity tests, where BW30 obtained a flux ranging from 31.49 to 90.66 L m^−2^ h^−1^, but when modified with the interphase polymerization method, it decreases from 24.02 to 77.01 L m^−2^ h^−1^ due to the newly added PA layer [9,22,24,26]. Similarly, the flux increased with the addition of ZnO NP, where the membrane that presented the highest value was the 10 mg ZnO NP with a range of 30.69 to 103.17 L m^−2^ h^−1^; additionally, this membrane was superior in terms of permeate flux, with an average of 9.5% for 20 Hz and 12.2% for 30 Hz compared to the BW30 membrane.

**Figure 10:**
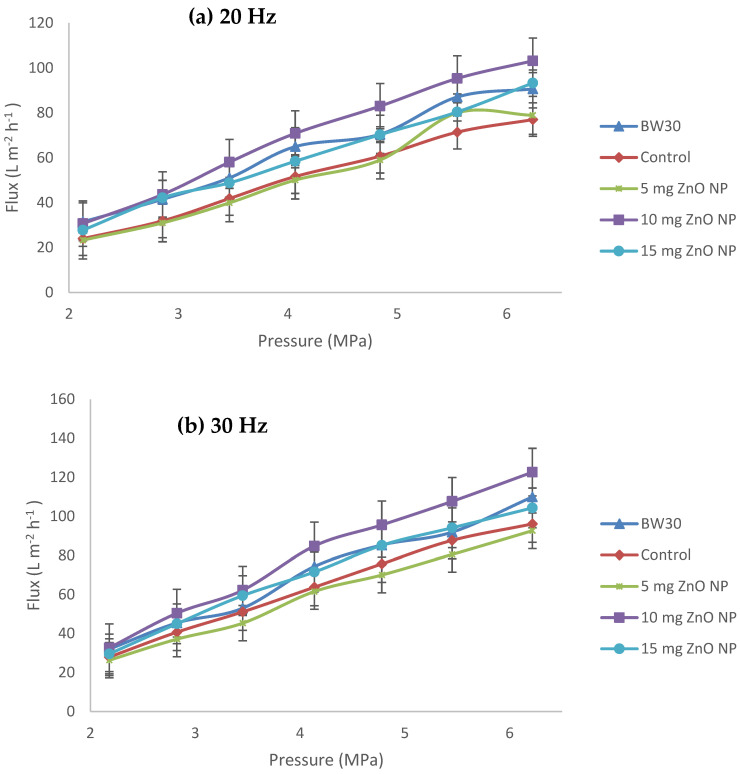
Pressure versus flux of the membranes: BW30, control, 5 mg ZnO NP, 10 mg ZnO NP, and 15 mg ZnO NP. (**a**) 20 Hz and (**b**) 30 Hz.

In contrast, for the 15 mg ZnO NP membrane, the flux decreased with the addition of the nanoparticles, obtaining 93.28 L m^−2^ h^−1^ at 6.2 MPa (Figure 10a). This behavior of increasing the amount of ZnO NP is also shown by Rabiee (2015). The permeate flux evaluated in his study reaches a maximum of 401.9 L m^−2^ h^−1^ at a concentration of 3% ZnO NP, but by increasing the concentration to 4%, the permeate flux decreased to 378.7 L m^−2^ h^−1^ [16]. These results coincide with the permeability and hydrophilicity tests and with several authors who report similar results [16,34,35,36], which is attributed to the agglomeration of nanoparticles [16,34,35,36]. The flux of the membrane with 5 mg ZnO NP, despite containing nanoparticles, is slightly higher than the control membrane; this is because it does not have enough NP to promote water diffusion through the semipermeable membrane, due to the formation of intermolecular forces of hydrogen bridges between the hydrogen in water and the oxygen in zinc oxide [27]. The graphs in Figure 10 of pressure versus permeate flux have a linear behavior, where the coefficient of determination (R^2^) has an average value in the five membranes of 0.987 for Figure 10a; in the case of Figure 10b, its average R^2^ is 0.991. The behavior in both graphs indicates that the permeate flux is directly proportional to the applied pressure with a linear behavior. According to Martinez-Perez (2023), “The correlation coefficient allows measuring the general agreement between two or more measurements involving quantitative variables, obtained with different measurement instruments or evaluators” [37,38].

**Figure 11:**
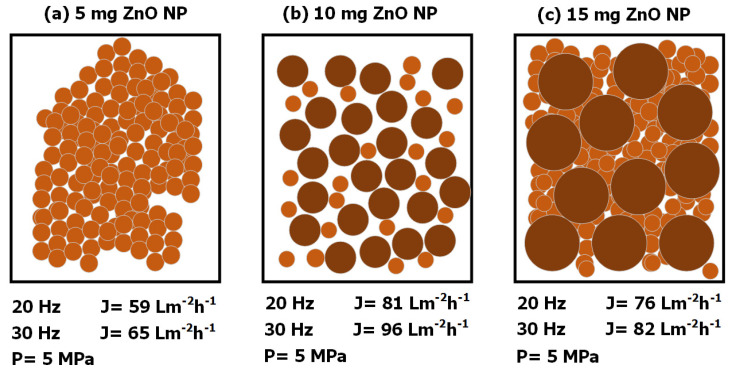
Distribution of nanoparticles on the surface of ZnO NP-modified membranes and their corresponding flux at 20 and 30 Hz and at an operating pressure of 5 MPa. (**a**) 5 mg ZnO NP, (**b**) 10 mg ZnO NP and (**c**) 15 mg ZnO NP.

With the SEM micrograph, a 2D scheme was elaborated (Figure 11) to explain that when we add a concentration of nanoparticles of 5 mg of ZnO distributed on the surface of a membrane, they are distributed in its nanometer scale, only they are not so well distributed and there is very little agglomeration, so the nanoparticles are very close together and obstruct the mass transport, and consequently, the flux is reduced [16,18,32]. When 10 mg of ZnO nanoparticles are added, there is a good distribution, but here there are more agglomerates and also nanoparticles with a size of 100 nm or less. This combination of nanoparticles and nanoparticle aggregates develops the perfect combination to create the conditions to have higher mass transport and increase the permeate flux [32,33]. It is assumed that the higher the concentration of nanoparticles, the higher the permeate flux, but in practice this does not happen. This is because there is a layer of nanoparticles deposited on the membrane surface and then another layer of agglomerates, which leaves little room for mass transport and therefore reduces permeate flux [17,18].

**Figure 12:**
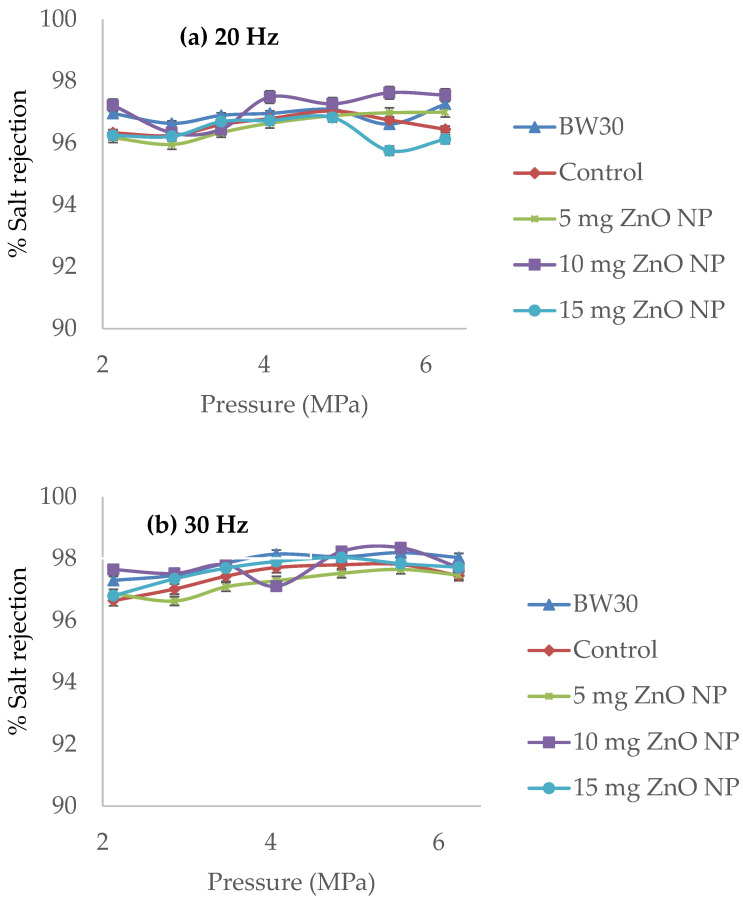
Pressure versus salt rejection of the membranes: BW30, control, 5 mg ZnO NP, 10 mg ZnO NP, and 15 mg ZnO NP. (**a**) 20 Hz and (**b**) 30 Hz.

Figure 12 shows the results of pressure versus salt rejection. In Figure 12a, the salt rejection ranges from 96% to a maximum of 97.55%; Isawi (2016) obtained very similar results with ZnO NP modified membranes from 96% to 98% salt rejection [18]. The membrane that performed best in terms of salt rejection at 20 Hz was the membrane with 10 mg ZnO NP with an average salt rejection of 97.13%. The BW30 membrane has an average salt rejection of 96.91%. In Figure 10b, the salt rejection ranges from 96.63% to a maximum of 98.35%. The membrane with the best performance at 30 Hz in terms of salt rejection was the BW30 membrane with an average salt rejection of 97.86% [18]. It is followed by the membrane with 10 mg ZnO NP; this has an average salt rejection of 97.77%, slightly lower than the BW30 membrane. The salt rejection by the membrane with 10 mg ZnO NP is sufficient to generate drinking water with an inlet of brackish water of 9000 ppm. Since the permeate would be 200.7 ppm, according to NOM-127-SSA1-2021, it can be used as drinking water [39]. The results summarized from highest to lowest percentage of salt rejection according to Figure 10a,b are as follows: 20 Hz: 10 mg ZnO NP > BW30 > control > 5 mg ZnO NP > 15 mg ZnO NP; and 30 Hz: BW30 > 10 mg ZnO NP > 15 mg ZnO NP > control > 5 mg ZnO NP. This indicates that the higher the pump operating frequency (30 Hz), the higher the permeate flux (Figure 10a,b).

**Figure 13:**
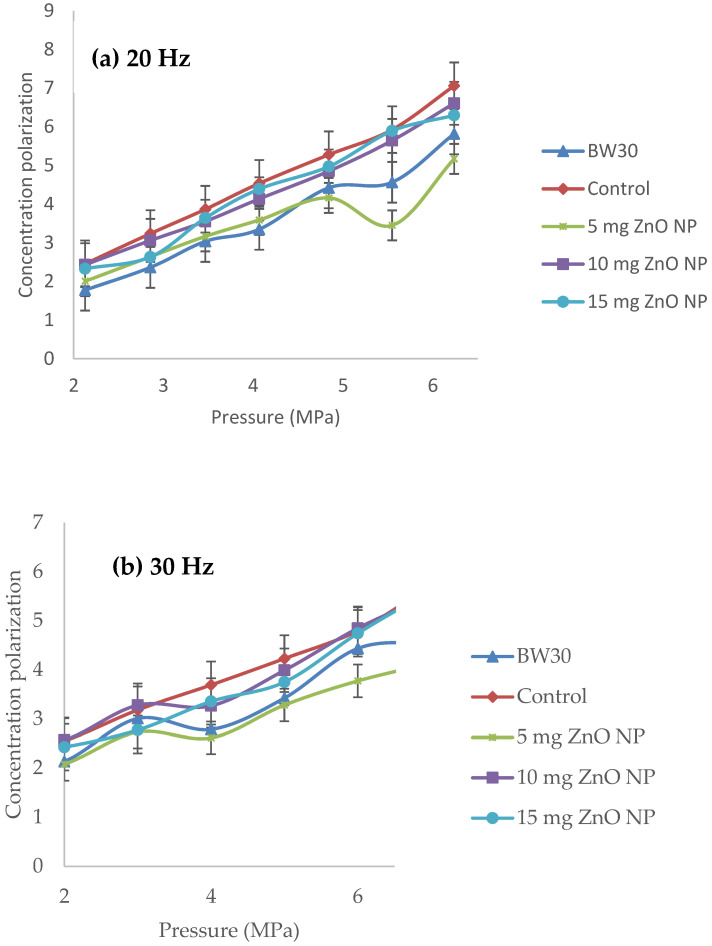
Pressure versus concentration of polarization of the membranes: BW30, control, 5 mg ZnO NP, 10 mg ZnO NP, and 15 mg ZnO NP. (**a**) 20 Hz and (**b**) 30 Hz.

Figure 13 shows the results of pressure versus concentration polarization. Plotting pressure versus concentration polarization in the cross-flow system shows a linear behavior, with the R^2^ at 0.9547 and 0.9654 at 20 Hz and 30 Hz, respectively [37,38]. The BW30 membrane and the membrane with 5 mg ZnO NP (Figure 13a) present the lowest concentration polarization. Contrasting with the results in Figure 12b, the membrane with 5 mg ZnO NP showed the lowest salt rejection, which indicates higher salt passage in the permeate; therefore, passing more salts polarizes the membrane less.

The membrane with 10 mg ZnO NP at 30 Hz (Figure 13b) showed the highest concentration polarization result, but this same membrane (Figure 12b) had higher salt rejections. Therefore, the higher the salt rejection, the higher the concentration polarization at the membrane surface, according to Ismail and Matsuura (2021) [6]. Thus, several authors, such as Kucera, 2015 and Dévora-Isiordia, 2023 [11,12,40], emphasize that the pressures for brackish water, as in this case of 9000 ppm, should be operated in a range of 1.8 ± 0.2 MPa in order to avoid problems of salt deposition in the active layer of the polyamide, as well as the negative effects of water hammer and clogging, by obstructing the flow through the membrane.

## 4. Conclusions

Modification of brackish water composite (BW30) membranes (TFC) with ZnO nanoparticles was achieved using the interphase polymerization technique to improve the permeate flux and its characterization. The FTIR signals show that a new polyimide layer was formed in the range of about 1617–1620 cm^−1^. The 1718.80 to 1716.15 cm^−1^ signal indicates that the -COOH functional group was formed. The contact angle corroborates that as the concentration of ZnO NP increases, the degree of hydrophilicity increases, i.e., the contact angle decreases. The 2D AFM and SEM results indicate that the nanoparticles were best distributed on the surface of the 10 mg ZnO NP membrane.

The membrane that performed best in terms of permeate flux was the membrane with 10 mg ZnO NP with an average permeate flux of 9.5% (103.17 L m^−2^ h^−1^ at 6.24 MPa) for 20 Hz and 12.2% (122.63 L m^−2^ h^−1^ at 6.24 MPa) for 30 Hz compared to the BW30 membrane (unmodified membrane). The membrane with 10 mg ZnO NP showed a salt rejection of 97.13% and 97.77% for 20 and 30 Hz, respectively, which are sufficient values to generate drinking water from 9000 ppm brackish water. The polarization results in the cross-flow equipment show a linear behavior, as the higher the operating pressure, the higher the concentration polarization will be above the recommended values. Therefore, it is advisable to find a balance between the flow of feed water at the industrial or laboratory level with respect to the key variables of the process, taking care that they do not exceed the operating limits recommended by the manufacturers.

## Figures and Tables

**Table 1:** Aqueous solutions.

Membranes	Solution B (Aqueous) (ZnO NP)
BW30	-
Control	-
5 mg ZnO NP	5 mg
10 mg ZnO NP	10 mg
15 mg ZnO NP	15 mg

- Indicates no use of ZnO NP.

**Table 2:** Equations for the calculation of the performance of desalination membranes.

Equation	Equation Number	Description and Reference
$z=\beta {\left[\frac{C}{1+\left(\alpha^{0}+\alpha^{1}\;z\right)\left(t-25{}^{\circ}C\right)\;}-Cz^{25}\right]}^{\gamma }$	(1)	Adjustment interactions between the conductivity of synthetic seawater and seawater [12]
$\begin{array}{c} \mu_{w}=\exp(5.495921\times 10^{5}T^{-2}\\ \;\;\;\;\;\;\;\;\;\;\;\;\;\;\;\;\;\;\;\;\;\;\;\;\;\;\;-1.66779\times 10^{3}\;T^{-1}\\ \;\;\;\;\;\;\;\;\;\;\;\;\;\;\;\;\;\;\;\;\;\;\;\;\;\;\;-7.612821) \end{array}$	(2)	Viscosity of distillated water [19]
$J_{v}=\frac{Q_{p}}{A_{m}}$	(3)	Permeate flux [6,7]
$\begin{array}{c} \mu =\mu_{w}(1+\sqrt{\omega }\left(b_{1}+b_{2}T^{3}\right)+\omega \left(b_{3}+b_{4}T^{3}\right)\\ +b_{5}\omega^{2}T^{3}) \end{array}$	(4)	Viscosity of the salt water. [20]
$\%R_{\mathrm{obs}}=\frac{C_{a}-C_{p}}{C_{a}}\times 100\%$	(5)	Observed salt rejection [6,7,12]
$⎾=\frac{\Delta P-J_{v}{\;\mu \;R}_{m}}{\;\left(C_{a}-C_{p}\right)}$	(6)	Polarization factor [12,21]
${\%R}_{\mathrm{int}}=\frac{1}{\;⎾\left(\frac{1}{R_{\mathrm{obs}}}-1\right)+1}\times 100\%$	(7)	Intrinsic rejection of salts [12,21]

**Table 3:** Infrared spectrum signals of the unmodified and modified membranes.

Functional Groups	Range 1/λ (cm^−1^)	1/λ (cm^−1^) BW30	1/λ (cm^−1^) BW30 Control	1/λ (cm^−1^) 5 mg ZnO	1/λ (cm^−1^) 10 mg ZnO	1/λ (cm^−1^) 15 mg ZnO
Carboxylic group R-COOH	1725–1700	-	1718.80	1718.18	1716.26	1716.15
Stretching –C=O (Amide III)	1680–1630	1659.90	-	-	-	-
Bending Vibration CO-NH (Amide I and II)	1640–1550	1609.44	1617.30	1616.87	1617.08	1619.88
Bending Vibration –CH_3_	1475–1365	1488.27 1458.96 & 417.25	& 1479.83 1438.08 & 1419.62	& 1483.15 1443.62 & 1419.20	&1473.39 1442.99 & 1416.33	&1471.88 1443.46 & 1418.01
Asymmetric stretching C-O-C of the aryl ether group	1300–1000	1244.60	1246.84	1245.16	1248.01	1247.25
Symmetric stretching of the sulfone functional group attached to aromatic rings (Ar-SO_2_-Ar)	1300–1000	1169.59 & 1151.22	1172.09 & 1157.16	1171.17 & 1152.93	1187.14 & 1151.59	1185.75 & 1152.34

- No signal was presented.

**Table 4:** Membrane roughness of the unmodified and modified membranes by AFM and micrometer.

Membrane	Average Roughness (nm) with Different Scanning Areas			Thickness (µm)
	10 µm	30 µm	50 µm	
BW 30	79.65	59.30	60.60	133.53 ± 3.84
Control	131.55	222.50	323.00	131.35 ± 1.24
5 mg ZnO NP	170.03	263.00	434.50	134.98 ± 3.42
10 mg ZnO NP	140.20	338.50	354.00	133.53 ± 2.48
15 mg ZnO NP	266.50	430.00	619.50	133.89 ± 1.24

## Data Availability

The original contributions presented in the study are included in the article, further inquiries can be directed to the corresponding author.

## Nomenclature

**Variables**	**Description**	**Units**
**C**	Electrical conductivity of water at the measured temperature. In conclusion, mean conductivity of synthetic seawater	(μS cm^−1^)
**z**	Adjustment interactions between the conductivity of synthetic seawater and seawater	(mg L^−1^)
**T**	The temperature at which conductivity was measured in the seawater.	(K)
**Q_p_**	Permeate flow rate	(m^3^ s^−1^)
**A_m_**	Membrane area	(m^2^)
**J_v_**	Flux	(m s^−1^)
**μw**	Viscosity	(Pa s)
**%R_obs_**	Salt rejection observed	(%)
**Ca**	Feed water concentration	(mg L^−1^)
**Cp**	Permeate water concentration	(mg L^−1^)
**R_m_**	Membrane resistance	(1 m^−1^)
**β**	Experimental constant with a value of 4.10 × 10^−7^ for salt from instant ocean sea salt.	
**α** **^₀^**	Constant for instant ocean sea salt of 0.0209026.	
**α** **^₁^**	Constant for instant ocean sea salt of 0.0347997.	
**ω**	Mass fraction of salt water in process.	
**b₁, b** **₂, b** **₃, b** **₄ y b** **₅**	Constants for solution viscosity calculation: −1.07266, 1.2722 × 10^−7^, −56.2241, 1.3332 × 10^−6^, 1.2053 × 10^−5^ respectively and were obtained experimentally with instant ocean sea salt.	
**TMC**	Synonyms: 1,3,5-Benzenetricarbonyl trichloride, Benzene-1,3,5-tricarbonyl chloride, Trimesic acid trichloride, and Trimesoyl chloride.	
** ⎾ **	Concentration polarization	
**Δ** **P**	Pressure change	
**%R_int_**	Intrinsic salt rejection	
